# A randomized phase II study to compare oxaliplatin plus 5-fluorouracil and leucovorin (FOLFOX4) versus oxaliplatin plus raltitrexed (TOMOX) as first-line chemotherapy for advanced colorectal cancer

**DOI:** 10.1007/s12094-012-0843-x

**Published:** 2012-07-19

**Authors:** Cristina Gravalos, Antonieta Salut, Carlos García-Girón, Rocío García-Carbonero, Ana Isabel León, Isabel Sevilla, Joan Maurel, Beatriz Esteban, Eduardo García-Rico, Adolfo Murias, Hernán Cortés-Funes

**Affiliations:** 1 Medical Oncology Department, University Hospital 12 de Octubre, Avda. de Andalucía s/n Km 5.400, 28041 Madrid, Spain; 2 Hospital Arnau de la Villanova, Lleida, Spain; 3 Hospital General Yagüe, Burgos, Spain; 4 Hospital Severo Ochoa, Leganés, Spain; 5 Fundación Jiménez Díaz, Madrid, Spain; 6 Hospital Virgen de la Victoria, Málaga, Spain; 7 Hospital Clinic i Provincial, Barcelona, Spain; 8 Hospital General de Segovia, Segovia, Spain; 9 Hospital de Monteprincipe, Madrid, Spain; 10 Hospital Insular de Las Palmas, Gran Canaria, Spain

**Keywords:** Colorectal cancer, Raltitrexed, Oxaliplatin, Fluorouracil

## Abstract

**Introduction:**

The aim of this study was to compare TOMOX versus FOLFOX4 as first-line treatment of advanced colorectal cancer (CRC).

**Materials and methods:**

191 chemotherapy-naïve patients were randomized to receive TOMOX or FOLFOX4. Patients were evaluated every 3 months and chemotherapy was continued until disease progression or unacceptable toxicity. Overall response rate was the primary endpoint.

**Results:**

183 patients were included in the intent-to-treat analysis (92 TOMOX and 91 FOLFOX4). Overall response rate was 45.6 and 36.3 % (*p* = 0.003) for TOMOX and FOLFOX4, respectively. No statistically significant differences were observed in overall survival (15.6 and 17.2 months; *p* = 0.475); progression-free survival (7.7 and 8.7 months; *p* = 0.292), and response duration (6.4 and 7.6 months; *p* = 0.372) for TOMOX and FOLFOX4, respectively. Grades 3 and 4 neutropenia (*p* < 0.0001) and leukopenia (*p* = 0.028) were more common with the FOLFOX4 regimen, while hepatic disorders and asthenia were higher in TOMOX group (*p* = ns). There were two treatment-related deaths in the FOLFOX4 arm and one in the TOMOX arm. Quality of life analysis based on the SF-36 revealed differences between the two regimens for physical and mental composite scores after 6 weeks, and for body pain and emotional role functioning after 6 and 12 weeks; all of these favored the FOLFOX4 arm (*p* ≤ 0.05).

**Conclusions:**

TOMOX and FOLFOX4 seem to have similar efficacy and are well tolerated in the first-line treatment for advanced CRC with different profiles of toxicity. The convenient TOMOX regimen may offer an alternative to fluoropyrimidine-based regimens.

## Introduction

Colorectal cancer (CRC) is the second most prevalent cancer worldwide, with more than 1 million new cases reported per year, accounting for 9.4 % of total cancers diagnosed globally [1]. In Europe, CRC is the second most commonly diagnosed cancer and accounts for 13 % of newly diagnosed cases [2]. Approximately 20–30 % of patients present with advanced disease. The prognosis for such patients is not promising [3], however, palliative chemotherapy increases overall survival (OS), progression-free survival (PFS) and improved quality of life (QoL) for patients with advanced CRC, compared with best supportive care [4, 5].

For more than 40 years, 5-fluorouracil (5-FU)-based chemotherapy was the only treatment with activity in CRC [6, 7]. However, during the last 10 years, a number of drugs have been demonstrated activity for advanced CRC. Oxaliplatin in combination with bimonthly 5-FU/leucovorin (LV) has been shown to be superior to 5-FU/LV in terms of response rate (RR) and PFS without having a negative impact on patients’ QoL [8]. Regimens based on the De Gramont schedule combined with oxaliplatin (FOLFOX) administrated every 14 days are considered to be standard options of chemotherapy for the treatment of advanced CRC [9, 10], but may be unsuitable for some patients owing to the toxicity profile and inconvenient administration as it requires a central venous catheter implantation.

Raltitrexed (Tomudex^®^) is a specific inhibitor of thymidylate synthase (TS). Raltitrexed enters cells via the reduced-folate carrier and is polyglutamated by folylpolyglutamate synthase, which increases intracellular retention and leads to prolonged TS inhibition, DNA fragmentation and cell death [11, 12]. The mechanism of action of raltitrexed differs from that of 5-FU and its serum terminal half life is longer (148–379 h) [13], which allows raltitrexed to be administered with an extended dosing interval, every 3 weeks [11].

As a single agent, raltitrexed has been extensively studied in four large comparative clinical trials that included more than 2,000 patients with advanced CRC [14–17]. In three of these studies, raltitrexed 3 mg/m^2^ every 3 weeks was compared with 5-FU or 5-FU/LV, no statistically significant differences were found in RR or survival outcomes between treatment arms [14–16]. In a fourth study, reported only as an abstract, a significantly longer median OS for 5-FU/LV was observed compared with raltitrexed at dosing of 3 or 4 mg/m^2^ [17]. Raltitrexed generally had an acceptable tolerability profile, and was associated with less leukopenia and mucositis/stomatitis than 5-FU/LV [14, 15, 17], although increased toxicity of raltitrexed compared with 5-FU/LV was reported in one study [16]. In the trial by Pazdur et al. [17] high rate of toxic death was observed when the dosing of raltitrexed was 4 mg/m^2^.

Young et al. evaluated patient preferences between raltitrexed and 5-FU-based chemotherapy regimens, regarding to adverse events and administration schedules in 82 patients with advanced CRC. Showing a similar efficacy, patients showed preferences for raltitrexed over other regimens, based its administration schedule (15-min intravenous every weeks) and/or side effects profile [18].

The extended dosing interval of raltitrexed, together with the different mechanisms of action of raltitrexed and oxaliplatin, has led to clinical interest in combining the two drugs. Raltitrexed in combination with oxaliplatin (TOMOX) has been evaluated in several preclinical and clinical studies [19–21]. Based on a phase I/II dose–escalation trial, the optimal dose of raltitrexed and oxaliplatin to be used in the TOMOX combination was established as oxaliplatin 130 mg/m^2^ combined with raltitrexed 3 mg/m^2^ every 3 weeks [19]. In phase II studies of this combination regimen, TOMOX has shown promising RR, survival and toxicity results [21–25]. These studies have provided evidence that the TOMOX combination may be effective and well tolerated in patients with advanced CRC, and have provided rationale for further evaluation of the regimen in the first-line setting.

This randomized phase II trial was designed to determine whether TOMOX is as effective as FOLFOX in the first-line treatment of advanced CRC.

## Materials and methods

### Patients

Patients were ≥18 years with advanced, histologically or cytologically confirmed, non-resectable metastatic CRC with bi-dimensionally measurable disease, life expectancy ≥3 months, Eastern Cooperative Oncology Group (ECOG) performance status ≤2, adequate bone marrow (platelet count ≥100,000/L, neutrophil count ≥2,000 cells/L, and hemoglobin level ≥9.0 mg/dL), renal [serum creatinine concentration <1.25× upper limit of normal (ULN) and creatinine clearance (CrCl) >65 mL/min] and hepatic (serum bilirubin level ≤1.5× ULN, aspartate amino transferase and alanine amino transferase ≤2.5× ULN, and alkaline phosphatase ≤5× ULN) function. Exclusion criteria included previous chemotherapy for advanced disease (or adjuvant chemotherapy ≤6 months before enrollment), treatment with an experimental drug within 4 weeks of inclusion, uncontrolled intercurrent disease, bone metastases as the only manifestation of the disease, any malignancy within 5 years of study entry (except for adequately treated non-melanoma skin cancer or in-situ cervical carcinoma) and grade ≥2 peripheral neuropathy according to National Cancer Institute Common Terminology Criteria for Adverse Events (NCI-CTC) version 2.0. Pretreatment assessments included complete medical history, physical examination, performance status, complete blood count, serum chemistry, electrocardiogram, and baseline measurement of tumor size based on tomography scans (CT). The study was carried out in compliance with the Declaration of Helsinki, Good Clinical Practice Guidelines, and all applicable local regulatory requirements. Signed informed consent was obtained from all patients.

### Study design

In this phase II, multicenter, open-label study, patients were randomized centrally in a 1:1 ratio to receive FOLFOX4 or TOMOX. Randomization was carried out using a four patient block randomization system at each study site, centrally administrated. FOLFOX4 was administered as previously described [8]: leucovorin 200 mg/m^2^, bolus 5-FU 400 mg/m^2^ plus 22-h continuous infusion of 5-FU 600 mg/m^2^ days 1–2, and oxaliplatino 85 mg/m^2^ day 1, every 2 weeks. Patients in the TOMOX group received raltitrexed 3 mg/m^2^ as a 15-min infusion, followed 45 min later by oxaliplatin 130 mg/m^2^ as a 2-h infusion, on day 1 of 3-week cycles. Raltitrexed dose and administration schedules were adjusted according to CrCl on day 1 of each cycle (CrCl >65 mL/min: 100 % of planned dose every 3 weeks; CrCl = 55–65 mL/min: 75 % of planned dose every 4 weeks; CrCl = 25–54 mL/min: 50 % of planned dose every 4 weeks). If raltitrexed administration was delayed, administration of oxaliplatin was also delayed.

Tolerability was evaluated at baseline and before each cycle. All toxicities graded according to the NCI-CTC version 2.0 except peripheral neuropathy that was evaluated according with the Sanofi classification. Dose adjustments and administration delays were evaluated based on the each patient’s most severe toxicity. In FOLFOX4 group, bolus and continuous infusion 5-FU was reduced to 300 and 500 mg/m^2^, respectively, if neutropenia, thrombocytopenia, diarrhea, stomatitis, or other toxicities grade (G) 3–4 occurred. Oxaliplatin was reduced to 65 mg/m^2^ in case of paresthesias associated with pain or functional less lasted during 7–14 days, and it was stopped if longer. In the TOMOX regimen, in case of neutropenia or thrombocytopenia G3 or G4, raltitrexed was reduced to 75 or 50 %, respectively, and oxaliplatin was administered at 100 mg/m^2^. Regarding non-hematological toxicities, in case of diarrhea, or stomatitis G2, G3 or G4 raltitrexed was reduced at 75, 50 % or omitted, respectively. Oxaliplatin was reduced to 100 mg/m^2^ in case of paresthesias associated with pain or functional less lasted during 7–14 days, and was stopped if longer.

In both groups, treatment was continued until progressive disease (PD), death, withdrawal of informed consent, or unacceptable toxicity. In patients who achieved a complete response, treatment was continued for a maximum of 6 months with or without oxaliplatin, depending on degree of cumulative neurotoxicity. Prophylactic anti-emetics were administered according to normal clinical practice. Routine use of a granulocyte colony-stimulating factor was not allowed.

QoL was assessed every 6 weeks using the short form-36 (SF-36) questionnaire.

The primary objective of the study was to demonstrate the non-inferiority of TOMOX when compared with FOLFOX4 in terms of Objective Response Rate (ORR). To avoid bias, response was evaluated according to RECIST every 3 months, regardless of the number of cycles administrated. The secondary objectives included OS, PFS, response duration, tolerability and QoL. OS and PFS were assessed from date of randomization until progression (PD), death or last follow-up. Response duration was defined as date of first response until PD, death or last follow-up.

### Statistical analysis

Determination of sample size was based on the primary endpoint. Based on previously published data, the ORR for both regimens was expected to be ~50 %. The study was designed to detect a 12 % as a maximum difference for non-inferiority in ORR between the two arms, using α of 0.05 and a β of 0.20. To achieve this, the number of patients required was 430 (215 per group). Intergroup comparisons of ORR ±95 % confidence intervals (CI) were conducted using a normal asymptotic one-sided *Z* test for proportions (non-inferiority) on independent samples. Univariate analyses of OS and PFS were conducted according to Kaplan–Meier estimates [26]. Comparisons between survival distributions were made by Cox proportional hazards model regressions, hazard ratios (HR) and 95 % CI [27]. Statistical significance was defined as *p* ≤ 0.05. The limit for non-inferiority was established in 12 % applied to the HR 95 % CI limits.

## Results

### Patients

**Fig. 1:**
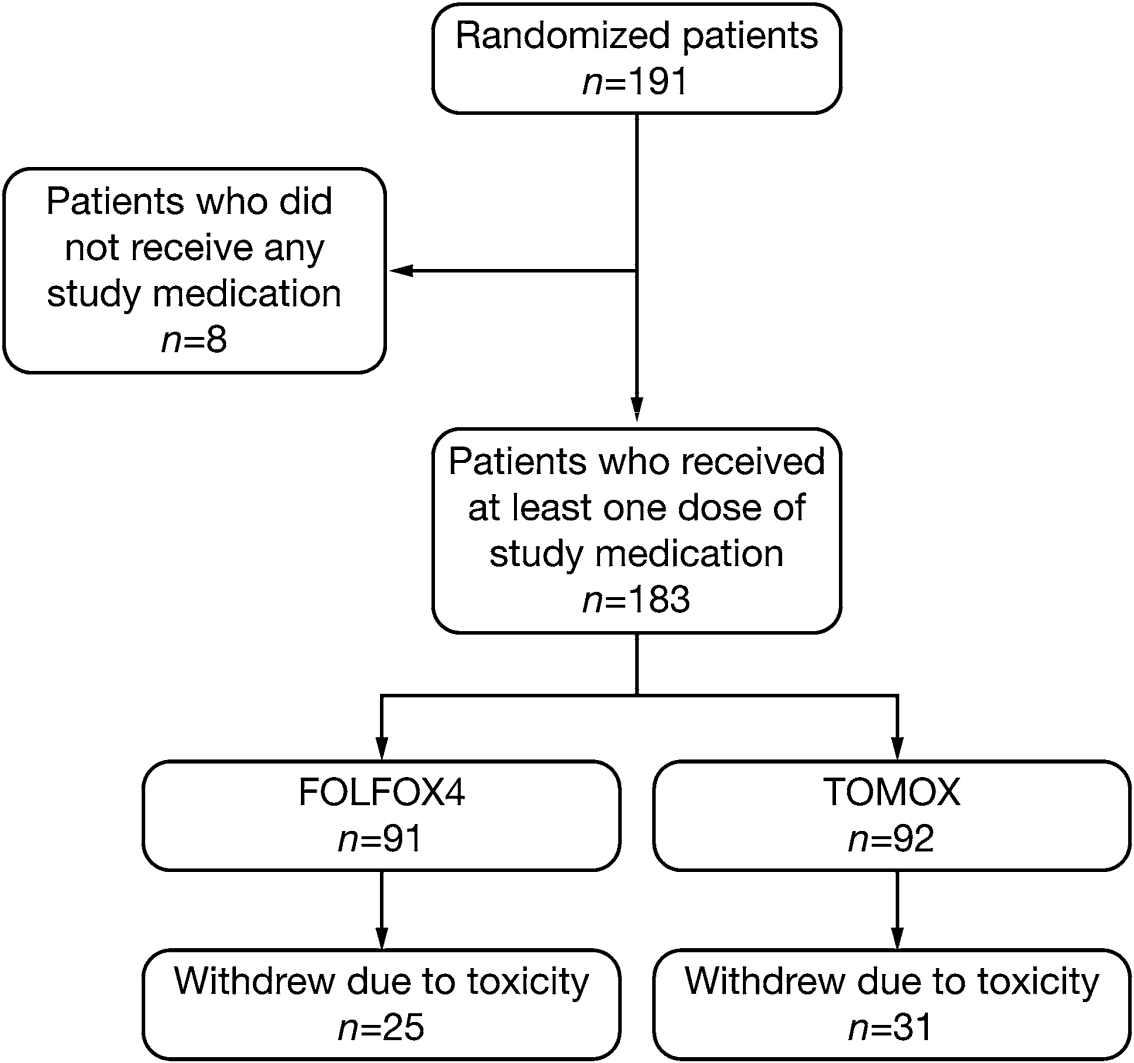
Disposition of patients

This phase II, multicenter, open-label, randomized study was conducted at 35 Spanish hospitals between January 2002 and February 2004. Due to limited funding, the study was closed prematurely for enrollment after 191 patients had been randomized. A total of 183 patients received at least one dose of study medication and were included in the intent-to-treat population (91 in the FOLFOX4 arm and 92 in the TOMOX arm; Fig. 1). Eight patients were excluded prior to receiving study medication due to; renal function out of range (2 patients), hematological function out of range (2 patients), hepatic and renal function out of range (2 patients) and for unknown reasons (2 patients).

Baseline demographics were similar between the two treatment groups, with no significant imbalances in sex, age, ECOG score or number of organs involved (Table 1). However, there was a significant difference between the groups with respect to location of primary tumor; there was a higher prevalence of rectal tumors in the TOMOX group than in the FOLFOX4 group (43.5 vs. 24.2 %; *p* = 0.005).

**Table 1:** Baseline characteristic of patients included in the study

Characteristic	Treatment group		*p* value
	FOLFOX4 (*n* = 91)	TOMOX (*n* = 92)	
Sex, *n* (%)			
Male	48 (52.7)	56 (60.9)	0.2674
Female	43 (47.3)	36 (39.1)	
Median age, years (range)	61 (35–82)	65 (36–78)	0.6542
Location of primary tumor, *n* (%)			
Colon	69 (75.8)	52 (56.5)	0.0058
Rectum	22 (24.2)	40 (43.5)	
ECOG score, *n* (%)			
0	48 (52.7)	51 (55.4)	0.5544
1	39 (42.9)	39 (42.4)	
2	4 (4.4)	2 (2.2)	
Number of organs involved, *n* (%)			
0	1 (1.1)	0 (0.0)	0.8765
1	59 (64.8)	64 (69.6)	
2	23 (25.3)	20 (21.7)	
3	8 (8.8)	7 (7.6)	
4	0 (0.0)	1 (1.1)	

*ECOG* Eastern Cooperative Oncology Group

The FOLFOX4 group received a median of eight cycles of treatment and the TOMOX group received a median of six cycles of treatment. Reasons for treatment withdrawal included toxicity (27.5 vs. 33.7 % of patients, respectively), and PD (28.6 vs. 28.3 % of patients, respectively). In the FOLFOX4 group, the median relative dose intensity was 84 % for both 5-FU and oxaliplatin. In the TOMOX group, the median relative dose intensity was 92 % for raltitrexed and 93 % for oxaliplatin.

### Efficacy

**Fig. 2:**
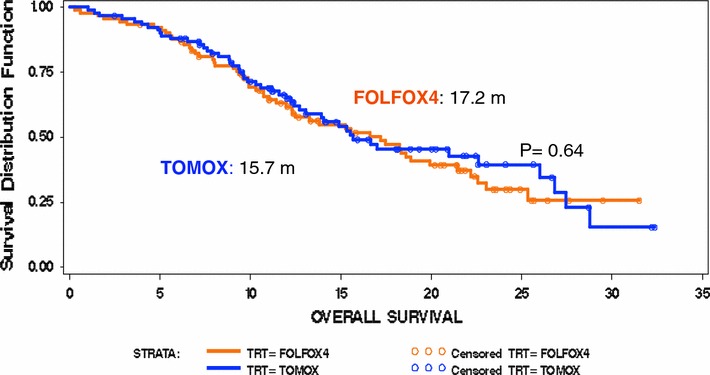
Kaplan–Meier estimates of overall survival

**Fig. 3:**
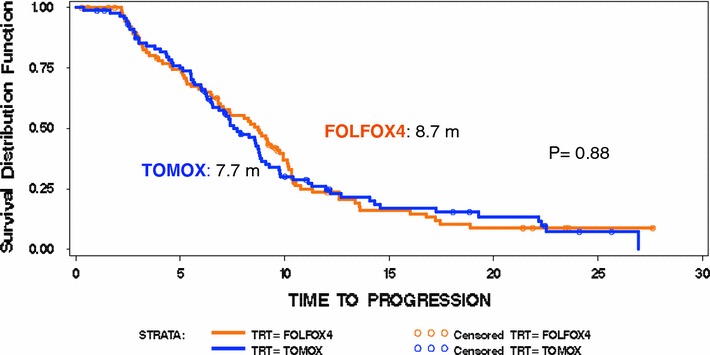
Kaplan–Meier estimates of time to disease progression

ORR was 36.3 % for FOLFOX4 and 45.6 % for TOMOX (*p* = 0.0032) (Table 2) and the non-inferiority of TOMOX in the primary endpoint when it is compared with FOLFOX4 was demonstrated. Disease control rate was similar for FOLFOX4 and TOMOX (69.3 and 74.9 %, respectively). With a median follow-up of 12.2 months, OS was 17.2 versus 15.7 months [HR 0.975 (95 % CI 0.655, 1.451; *p* = 0.475)] (Fig. 2), PFS was 8.7 versus 7.7 months [HR 0.927 (95 % CI 0.65, 1.292; *p* = 0.292)] (Fig. 3), and response duration was 7.6 versus 6.4 months (*p* = 0.372) for FOLFOX4 and TOMOX, respectively.

**Table 2:** Response evaluation

Response, *n* (%)	Treatment group	
	FOLFOX4 (*n* = 91)	TOMOX (*n* = 92)
Complete response	7 (7.7)	4 (4.3)
Partial response	26 (28.6)	38 (41.3)
Stable disease	30 (33.0)	27 (29.3)
Progressive disease	16 (17.6)	12 (13.1)
Not evaluable	12 (13.1)	11 (12.0)

*p* = 0.0032 for non-inferiority of TOMOX considering overall response (complete response + partial response)

### Safety

All patients, who received at least one dose of study medication were evaluable for toxicity assessments. The most common grades 3–4 adverse events (AEs) are summarized in Table 3. Grades 3–4 hematologic AEs were more frequent in the FOLFOX4 group than the TOMOX group [neutropenia: 34.1 vs. 5.4 % (*p* < 0.0001); leucopenia: 7.7 vs. 1.1 % (*p* = 0.028); thrombocytopenia: 6.6 vs 1.1 % (*p* = 0.064)]. Hepatic disorders (25.0 vs. 14.3 %) and asthenia (19.6 vs. 11.0 %) had a numerically higher incidence in the TOMOX group than the FOLFOX4 group, but the differences did not reach statistical significance. Regarding neurotoxicity, incidence of paresthesias grade >2 was similar for FOLFOX and TOMOX groups, (7.7 and 6.5 %, respectively). Sixteen patients (18.6 %) in the FOLFOX4 group and 12 patients (13.0 %) in TOMOX group experienced serious AEs (SAEs). The difference in the incidence rate of SAEs was not statistically significant (*p* = 0.393). There were two treatment-related deaths in the FOLFOX4 group (one due to neutropenic sepsis; one due to pancitopenia plus septic shock) and one in the TOMOX group (due to septic shock).

**Table 3:** Grades 3 and 4 hematologic and non-hematologic toxicities

Adverse events, % patients	Treatment group		*p* value
	FOLFOX4 (*n* = 91)	TOMOX (*n* = 92)	
Thrombocytopenia	6.6	1.1	0.064
Leukopenia	7.7	1.1	0.028
Neutropenia	34.1	5.4	<0.0001
Diarrhea	11	9.8	NS
Nausea/vomiting	11	6.5	NS
Asthenia	11	19.6	NS
Hepatic disorders	14.3	25	0.068
Paresthesia	7.7	6.5	NS

*NS* non-statistical significance

### Quality of life

A total of 161 patients (88 %) were included in the QoL assessment. At baseline, there were no significant differences in scores for either composite physical/mental health measurements, or any individual components of SF-36, between the two groups. However, significant differences in SF-36 scores between the groups emerged during treatment. Both composite physical health and mental health scores were lower in the TOMOX group than the FOLFOX4 group after 6 weeks of treatment [changes in mean physical health score from baseline: +4.1 for FOLFOX4 and −2.93 for TOMOX (*p* = 0.03); and changes in mean mental health score from baseline: +3.7 for FOLFOX4 and −2.1 for TOMOX (*p* = 0.05)]. There were no differences in composite scores after 6 weeks of treatment.

With respect to individual components of SF-36, there were significant differences in bodily pain and emotional role functioning scores in favor of FOLFOX4 after 6 and 12 weeks [bodily pain: +13.7 vs. +2.1 after 6 weeks (*p* = 0.05); +15.9 vs. +1.85 after 12 weeks (*p* = 0.01); emotional role functioning: +13.9 vs. −4.2 after 6 weeks (*p* = 0.01); +7.1 vs. −11.9 after 12 weeks (*p* = 0.05)].

## Discussion

Over the last decade, several phase II clinical trials have assessed TOMOX in patients with metastatic CRC and have demonstrated promising results in terms of ORR, survival and tolerability [21–25, 28–30]. However, few head-to-head studies have been published that compare TOMOX with established standard of care. In this study, first-line FOLFOX4 and TOMOX showed comparable efficacy in advanced CRC with acceptable tolerability profiles. These findings suggest that TOMOX could potentially be considered as a treatment option for patients with advanced CRC.

The efficacy of TOMOX in advanced CRC observed in the current study was in accordance with previously published findings [21–25, 28–30]. The efficacy outcomes for the FOLFOX4 arm are in line with those achieved by De Gramont [8]. The median PFS reported by De Gramont et al. was 9 months, which is similar to the 8.7 months seen in our study. In contrast, the ORRs achieved in our study are lower (36.3 vs. 50.7 %). In the TOMOX arm of our study, the ORR, PFS and OS are within the general range obtained in previous trials. In three phase II clinical trials where patients with advanced CRC were treated with TOMOX, ORRs of 43 % [24], 46 % [28] and 54 % [21] were reported, compared to 45.7 % in our study. The median PFS values reported in the three studies (6.2, 8.2 and 10.3 months, respectively) are also in line with the 7.7 months observed in our study. Similarly, the median OS of 15.7 months in this study is comparable to the 14.6 and 14.5 months reported for two of these studies [21, 24]. Furthermore, in a phase III clinical trial that compared TOMOX with oxaliplatin plus 5FU/LV in 216 patients [31], the TOMOX arm was superior in terms of ORR (29.1 vs. 17 % *p* = 0.0437) although the oxaliplatin plus 5FU/LV arm ORR was lower than previously published.

Both regimens were well tolerated, although there were some differences in the safety profiles of FOLFOX4 and TOMOX in this study. As expected, the incidence of neutropenia and leukopenia grade was higher in the FOLFOX arm and the incidence of hepatic disorders and asthenia was higher in the TOMOX arm, although without statistical significance. The number of treatment-related deaths was similar in both treatment arms (2 in FOLFOX arm vs. 1 in the TOMOX arm), and no unexpected AEs occurred. These data suggest that raltitrexed may be a tolerable treatment for advanced CRC, provided dose level and schedules are adjusted according to changes in CrCl and in response to the emergence of hematologic and non-hematologic toxicities. When compared with the data published from the PETACC1 study [32], the use of raltitrexed in our study did not increase the number of treatment-related deaths.

In contrast to 5-FU, which requires a 22-h infusion for 2 consecutive days every 2 weeks and a central venous device, raltitrexed is administered as a 15-min intravenous infusion every 3 weeks. Given that TOMOX does not seem to be associated with any additional safety and tolerability concerns compared with FOLFOX4, the relative convenience of raltitrexed administration may make TOMOX an attractive option for first-line treatment of patients with advanced CRC, particularly in patients who find it difficult to meet the scheduling commitments for FOLFOX4 infusions, who cannot tolerate 5-FU-based regimens, or who cannot have a central venous catheter [33, 34].

Our study was limited because of the sample size estimated in the statistical plan was not achieved due to funding issues. However, the overall response rate, main endpoint of the study, was not affected and the results demonstrate the non-inferiority of TOMOX treatment when it is compare with FOLFOX4. There is a lack of power to reach significance in outcomes related to survival even though all HR point estimates were very close to 1, which indicates similarity between both regimens, and the non-inferiority of TOMOX could not be proven for these secondary endpoints.

Finally, it is important to note that several monoclonal antibodies have been approved for clinical use in patients with advanced CRC: the antivascular endothelial growth factor antibody bevacizumab and the anti-epidermal growth factor receptor antibodies cetuximab and panitumumab. However, targeted therapies were not standard treatment options when the current study was running. Further studies will be required to guide the optimal application of TOMOX in relation to the range of other treatment options now available for advanced CRC.

In conclusion, our study indicates that the TOMOX regimen appears similar to FOLFOX in terms of efficacy and tolerability as first-line treatment for advanced CRC. Furthermore, the ambulatory administration schedule could provide greater convenience for a large number of patients. However, confirmatory phase III studies are required to fully establish the efficacy and safety profile of the regimen. Combinations of TOMOX with monoclonal antibodies require investigation in clinical trials.
